# Comparative genomics reveals details into the metabolism of peritrich ciliates (Ciliophora, Oligohymenophorea and Peritrichia)

**DOI:** 10.1099/mgen.0.001472

**Published:** 2025-09-04

**Authors:** Pedro Mendes de Souza, Franciane Cedrola, Roberto Júnio Pedroso Dias, Vera Nisaka Solferini

**Affiliations:** 1 Laboratório de Protozoologia, Departamento de Zoologia, Universidade Federal de Juiz de Fora, Juiz de Fora, Minas Gerais, Brazil; 2 Laboratório de Diversidade Genética, Departamento de Genética, Evolução, Microbiologia e Imunologia, Instituto de Biologia, Universidade Estadual de Campinas, Campinas, São Paulo, Brazil; 3 Center for Computational Engineering and Sciences, Universidade Estadual de Campinas, Campinas, São Paulo, Brazil

**Keywords:** carbohydrate metabolism, functional annotation, Peritrichia, phylogenomics

## Abstract

The subclass Peritrichia encompasses over 1,000 species of ciliates, demonstrating both wide distribution and significant morphological diversity across aquatic environments. Despite their ecological significance and unique biological attributes, genomic information for peritrichs has remained sparse. This study aimed to fill this gap by sequencing the genomes of seven distinct species of peritrich ciliates and employing advanced genomic technologies to investigate their metabolic characteristics, functional diversity and evolutionary relationships. Our analyses revealed a large number of genes annotated across various functional categories, with EggNOG annotations highlighting genes related to post-translational modification, translation and energy metabolism, suggesting a high adaptive and metabolic capacity. Notably, the diversity of CAZymes, particularly glycoside hydrolases, indicates metabolic complexity and the ability to degrade carbohydrates, critical for environmental adaptation and bacterial predation. Additionally, the presence of chitin-degrading enzymes in species like *Trichodina acuta* points to complex ecological interactions with hosts, with potential implications for natural ecosystems and commercial hatcheries. A concentration of glycosyl transferases found in the Vaginicolidae family suggests an enhanced capacity for glycoconjugate synthesis, possibly linked to lorica formation. The phylogenomic reconstruction was consistent with existing literature, uncovering intricate phylogenetic relationships. Overall, our findings significantly advance the understanding of peritrich ciliates by shedding light on their metabolic potential, ecological adaptability and evolutionary complexity, while also providing a genomic foundation for future ecological and evolutionary studies.

**Impact Statement**

This study provides the first comprehensive genomic insight into the subclass Peritrichia, a diverse and ecologically significant group of ciliates. By sequencing and analysing the genomes of seven species, we unveil the metabolic versatility, ecological interactions and evolutionary complexity of these protists. Our findings highlight key functional adaptations, including carbohydrate metabolism and host-associated enzyme systems that shape the diversity and ecological roles of peritrich. This genomic framework lays the foundation for future research on protist biology, with implications for ecosystem dynamics and aquaculture health.

## Data Statement

All supporting data, code and protocols have been provided within the article or through supplementary data files.

## Introduction

The subclass Peritrichia (Ciliophora and Oligohymenophorea), comprising over 1,000 described species [1] and divided into more than 12 families, represents ~10% of the known diversity within the phylum Ciliophora [2]. Species within this subclass exhibit notable morphological diversity and are globally distributed, occupying a wide variety of aquatic environments [1,3]. Since their discovery by Leeuwenhoek in the seventeenth century, several distinctive features of peritrichs have been investigated, including their contraction mechanism – one of the fastest, surpassing the speed of a blink multiple times [4,6] – and their relevance as bioindicators, owing to their pronounced sensitivity to environmental changes and well-documented association with organic pollution [7].

Despite the scientific interest in this group, many knowledge gaps persist, particularly regarding their internal relationships, metabolic pathways and evolutionary history [8,10]. One of the primary challenges is that many peritrichs are difficult to isolate and culture, which complicates various types of biological investigation [11,12]. These difficulties are even more pronounced in molecular and genomic analyses, as many species live as epibionts on other organisms [2,3], increasing the risk of contamination and hindering data collection. Consequently, genomic information on this group remains scarce [9,13, 14], with only a few genomes currently deposited in the GenBank database – some of which have not yet been identified at the species level [8,9, 13,15].

Nevertheless, the limited genomic data available have already yielded insights into the group’s internal relationships [8,9], environmental adaptations [14] and even structural cell features relevant to taxonomy [10]. However, certain aspects of peritrich biology, such as feeding metabolism, remain poorly understood from a genomic standpoint. Most peritrichs are considered bacterivorous [3,16, 17], some are algivorous [4,17, 18] and a few exhibit parasitic lifestyles [19,21]. Yet, a comprehensive understanding of their functional roles in ecosystems — and, by extension, their full metabolic potential — remains largely unexplored.

To address these gaps, this study sequenced the genomes of seven peritrich ciliate species. Using genomic tools, we investigated their metabolic characteristics and performed a comparative analysis with previously described species. Furthermore, a phylogenomic analysis was conducted to propose a hypothesis regarding the evolutionary history of the subclass Peritrichia.

## Methods

### Sample collection, taxonomic identification, DNA extraction, amplification and sequencing

We obtained samples from seven peritrich ciliate morphospecies (*Epistylis anastatica*, *Platycola decumbens*, *Vorticella* sp. 3, *Telotrochidium* sp., *Thuricola similis*, *Trichodina acuta* and *Zoothamnium parahinketes*) isolated from distinct environments (Material S1, available in the online Supplementary Material), from soil to oceanic waters (BioProject number PRJNA1260544). The ciliates were screened from the samples with glass micropipettes, under a stereomicroscope and immediately fixed in absolute ethanol; a small sample of each morphospecies was preserved in Bouin and distilled water, for morphological characterization and taxonomic confirmation. Only individuals with a clear internal content were chosen for DNA extractions.

Pools of 30 ciliates of each morphospecies were used for total DNA extraction using the DNeasy Blood and Tissue Kit (QIAGEN), following the animal tissue protocol. Genomic DNA was amplified using the REPLI-g Single Cell kit (QIAGEN), and the product was sent to CD Genomics Service Company (https://www.cd-genomics.com/) for sequencing. The macronuclear genome of each morphospecies was sequenced using short reads (2×150 bp, Illumina MiSeq).

### Genome assembly

We assembled seven newly sequenced genomes and acquired Oligohymenophorea ciliates genomes/transcriptomes available in the Sequence Read Archive (SRA) NCBI (National Center for Biotechnology Information). Further information on genomes/transcriptomes obtained was provided in the S2.

The SRA genomes/transcriptomes were retrieved using the *fastq-dump* function available in the NCBI SRA toolkit with the following parameters (--clip --split-files --skip-technical). After inspection using FastQC v.011.9 (http://www.bioinformatics.babraham.ac.uk/projects/fastqc/), we performed a quality control protocol with each genome/transcriptome in fastp [22], using default parameters. Only reads with a *Q* score >20 per each base were selected for subsequent analyses. Following the quality control, the genomes were assembled using MEGAHIT v. 1.0 [23] (megahit −1–2 -o) and transcriptomes using TRINITY v. 2.8 [24], with default parameters. Bacterial genomes were downloaded from GenBank as blast databases to remove contamination. Considering that the G+C content of ciliates is relatively low, we excluded contigs with a G+C content higher than 50 mol% since they are likely to be contaminants, as suggested by a previous study [25]. Assembling statistics were obtained using QUAST v. 5.0.2 [26] with default parameters. BUSCO v. 4.0.6 [27] against the Alveolata OrthoDB v. 10 [28] was used to estimate overall genome completeness, also with default parameters.

Given the absence of physical separation between macronucleus and micronucleus prior to sequencing, it is expected that the genomic data contain a mixture of both nuclear types. However, due to the substantial ploidy difference, most of the sequencing data originates from the macronucleus. Therefore, we proceeded with downstream analyses using the full genomic assemblies, which are predominantly macronuclear but may include a small fraction of micronuclear sequences.

### Gene prediction and annotation and orthogroup analysis

Genes were predicted in each genome using AUGUSTUS v. 3.4, trained with the species *Tetrahymena thermophila* as a model [29]. Functional annotation was performed with EggNOG-mapper v. 2.0 [30,31] using the DIAMOND mapping mode [31] and against three functional databases: Cluster of Orthologous Genes (COG), Gene Ontology (GO) and Kyoto Encyclopedia of Genes and Genomes (KEGG). Carbohydrate-active enzymes (CAZymes) were annotated using the dbCAN3 meta server [32] against the CAZy database v. 9.0, which is a dedicated database for genomic, structural and biochemical analysis on CAZymes.

The OrthoFinder v. 2.5.2 [33] software was used to identify, among the protein coding sequences recovered in AUGUSTUS analyses, specific orthogroups for the class Oligohymenophorea, subclass Peritrichia, and Peritrichia internal clades (Epistylididae, Vaginicolidae, Vorticellidae, Zoothamniidae and Trichodinidae) – recovered in phylogenomic reconstruction (see ‘Phylogenomic reconstruction’ section).

### Phylogenomic reconstruction

A phylogenomic analysis was performed using 32 Oligohymenophorea genomes/transcriptomes, using OrthoFinder. Orthofinder assigned 1,418,531 genes (72.2% of the total) to 203,666 orthogroups. Fifty per cent of all genes were in orthogroups with 6 or more genes (G50 was 6) and were contained in the largest 42,345 orthogroups (O50 was 42,345). A gene tree was inferred based on each orthogroup using -M msa -T fasttree parameter; a rooted species tree was estimated from all inferred gene trees using STAG and STRIDE methods [34,35], both implemented in OrthoFinder.

## Results

### Genome assembly

The newly predicted genomes varied greatly after assembly. The assembly size varies from 29.32 to 85.02 Mb, as estimated from 2,827 to 79,758 contigs assembled (G+C content 25.02–46.29 mol%; N50 2,294–5,6673 bp; L50 162–6,365 bp), exhibiting a completeness of 45.6–73.1, as indicated through BUSCO analyses. Details on assembly metrics are presented in Table 1.

**Table 1.** Genomic features of peritrich ciliates sequenced

Species	No. of contig	Total length (bp)	Total length (Mb)	Largest contig	G+C content (mol%)	N50 (bp)	N90 (bp)	L50 (bp)	L90 (bp)	Genome completeness (%)
*Epistylis anastatica*	59,868	84,591,044	84.59	172,289	25.02	3,717	721	3,871	25,293	63.8
*Thuricola similis*	51,292	67,689,519	67.69	94,159	27.42	7,128	716	1,899	15,228	45.6
*Trichodina acuta*	77,876	85,020,461	85.02	190,022	42.44	3,008	646	3,310	27,997	64.9
*Platycola decumbens*	49,224	58,060,504	58.06	199,022	32.41	11,288	643	716	13,851	51.4
*Vorticella* sp.	49,313	63,029,759	63.03	144,737	46.29	5,933	672	2,024	16,742	73.1
*Telotrochidium* sp.	2,827	29,317,743	29.32	181,229	32.24	56,673	15,211	162	526	59.7
*Zoothamnium parahinketes*	79,758	82,448,287	82.45	83,390	37.03	2,294	643	6,365	31,654	66.1

### Orthogroups analyses and phylogenomic reconstruction

**Fig. 1.**
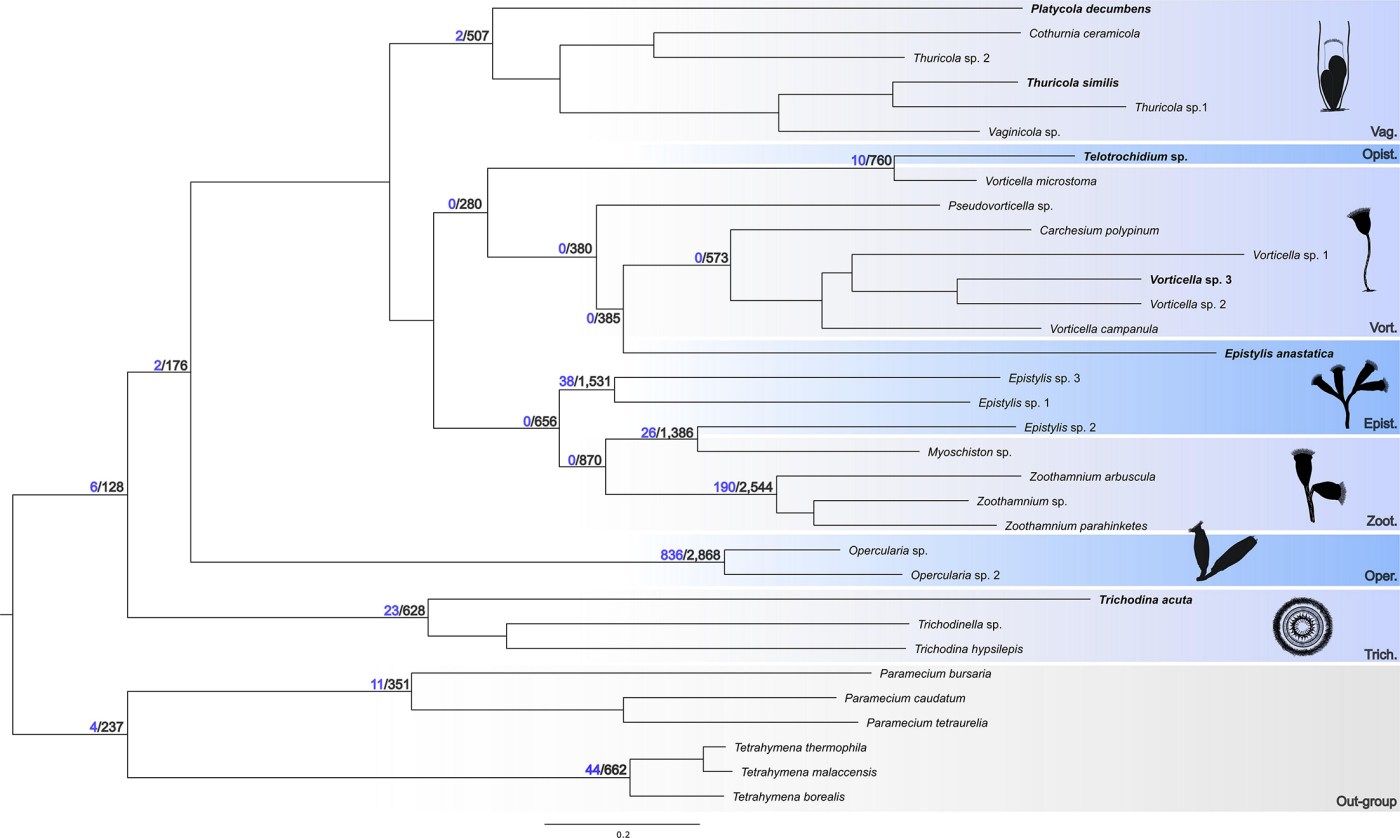
Phylogenomic tree constructed based on orthogroup gene trees. Black numbers indicate the number of shared orthogroups for each clade, while blue numbers specify the number of unique orthogroups exclusive to each clade. Vag., Vaginicolidae; Opist., Opisthonectidae; Vort., Vorticellidae; Epist., Epistylidae; Zoot., Zoothamnidae; Oper., Operculariidae; Trich., Trichodinidae.

A total of 203,666 orthogroups were identified across Oligohymenophorea genomes and transcriptomes. Phylogenomic analysis (Fig. 1) positioned non-peritrich species (*Paramecium* and *Tetrahymena*) as a sister group to all peritrichs. Oligohymenophorea species shared 94 orthogroups, with the *Paramecium*/*Tetrahymena* clade sharing 237, including 4 unique to this group. The Peritrichia clade had 128 shared orthogroups, with 6 being unique, and formed 2 monophyletic groups: Sessilida and Mobilida. Sessilida had 176 orthogroups, Mobilida had 628 and various families within these orders, such as Opercularidae and Vaginecolidae, also showed shared and unique orthogroups.

### Gene prediction and functional annotation

**Fig. 2.**
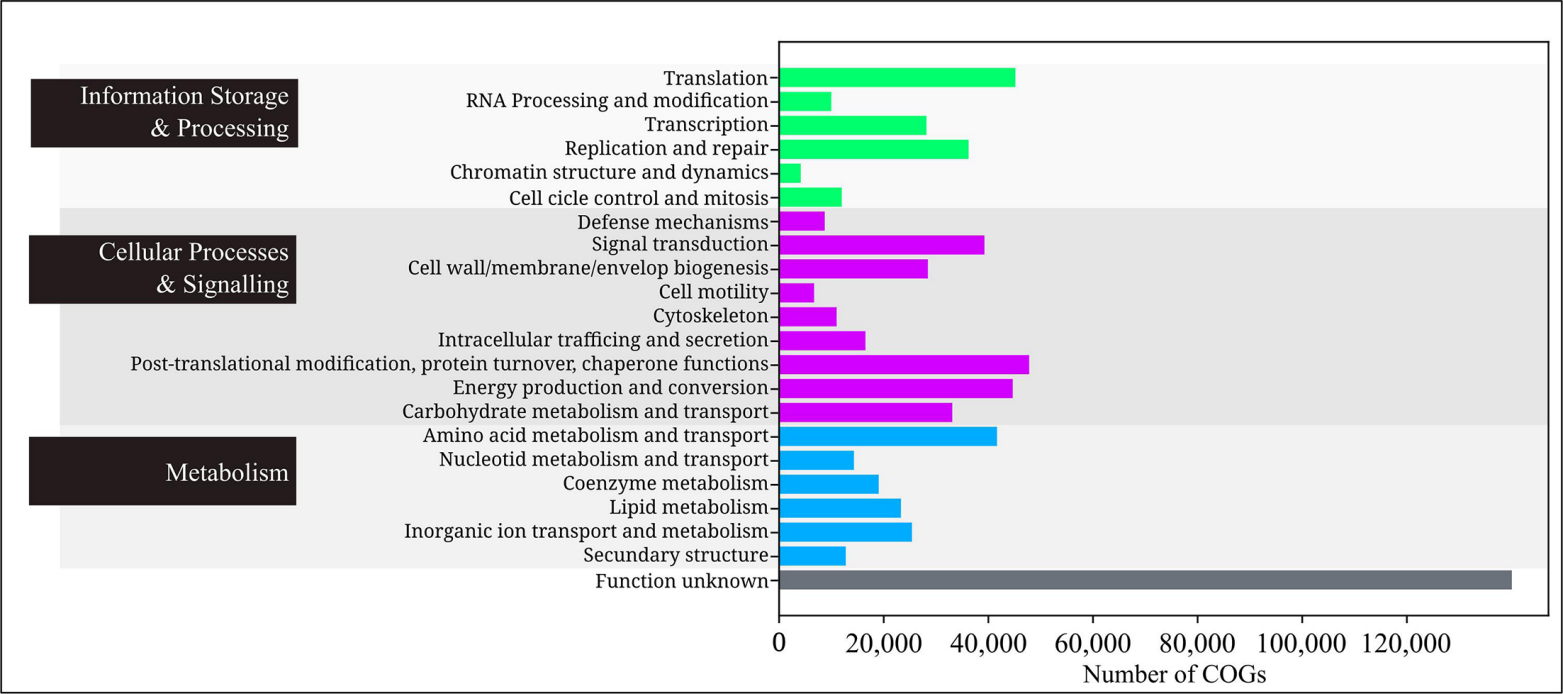
Bar graph showing the number of COGs found for all the genomes analysed for each of the COG categories.

No significant differences were observed in the general annotations across taxonomic groups. From 1,963,490 genes annotated, 648,245 genes were categorized into 23 COG categories, with 'Post-translational Modification' being the most represented, followed by categories ‘Translation’, ‘Energy production and conversion’ and ‘Amino acid metabolism and transport’ (Fig. 2).

GO annotations assigned 1,032,935 genes to 113 non-redundant terms, with ‘Biological Processes’ being the most prevalent, comprising on average 24% of the total terms, followed by ‘Metabolism’ (7%) and ‘Molecular Function’ (5%).

KEGG annotations mapped 429,002 genes to 13,960 functional orthologs across 486 pathways, involved in ‘Metabolism‘, ‘Genetic Information Processing’, ‘Environmental Information Processing’, ‘Cellular Processes’, ‘Organismal Systems’, ‘Human Diseases’ and ‘Drug Development’.

Detailed information regarding EggNOG functional annotations for each species can be found in the Material S3.

**Fig. 3.**
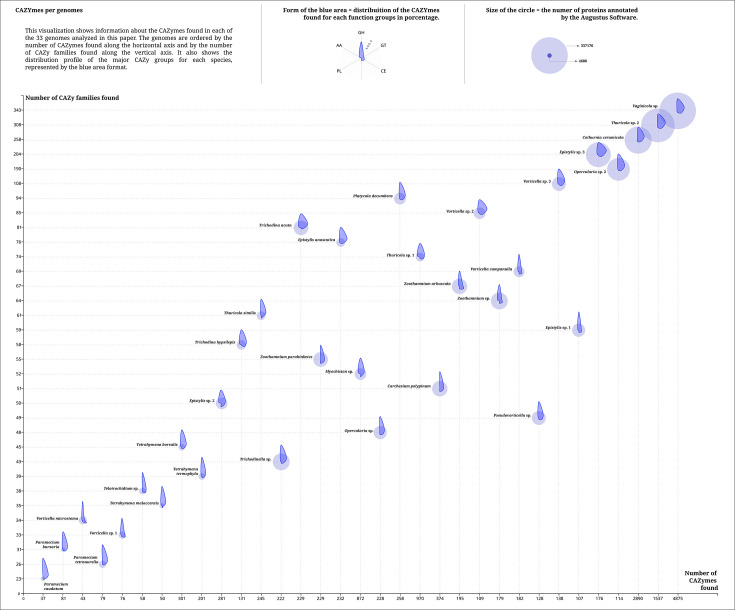
Scatter plot showing the variation in number of proteins recovered, number of CAZymes annotated and CAZyme family distribution profile for each of the species analysed.

Additionally, CAZyme analysis identified 15,827 enzymes across 476 families of glycoside hydrolases (GHs), carbohydrate-binding modules (CBMs), carbohydrate esterases (CEs), glycosyl transferases (GTs), and auxiliary activities (AAs), involved in carbohydrate breakdown and synthesis (Fig. 3).

*Paramecium* and *Tetrahymena* species had 435 CAZymes, mostly GHs, 300 (~69%), followed by GTs, 77 (~18%), with *Paramecium* having fewer enzymes than *Tetrahymena*. The most common families for these genera were GH31_1, GT2, GH38, CE13 and GH16_3, which act, respectively, in non-structural, unknown, non-structural, microbial cell-wall and structural carbohydrates.

For the order Sessilida, 14,950 CAZymes were annotated. Most of the CAZyme families found were GHs, 7,431 (~50%), followed by GT, 3,380 (~22,5%). For the whole order, the most frequent families were GT2, GT4, CE1, GH177 and CE13, which act, respectively, in unknown, unknown, microbial cell-wall, non-structural and microbial cell-wall carbohydrates.

Within Sessilida, we found some different profiles in the CAZyme distribution. The family Vaginicolidae, although also having the GH families as more abundant, followed by the GT families, shows a different ratio than the other taxonomic groups. The GH families represent close to 46.5% of the enzymes found and the GT groups 23%. This profile is not common in other clades such as Vorticellidae (~59% GH to ~20.5% GT) and Epistylididae/Zoothamniidae (~55.5% GH to ~21% GT). As for the most abundant CAZyme families for each clade found, we have CE13, GT2, GT4, CBM13 and GH20 acting in microbial cell-wall, unknown, unknown, unknown and structural carbohydrates for Epistylididae/Zoothamniidae. We have GT4, GT2, CE1, GH177 and GH179 acting in unknown, unknown, microbial cell-wall, non-structural and structural carbohydrates for Vaginicolidae and lastly CE13, GT2, GH18, GH20 and GH31_1 acting in microbial cell-wall, unknown, microbial cell-wall, structural and non-structural for Vorticellidae. The CAZy family GT2 is the only one in high numbers in the three recovered clades; CE13 and GH20 were found in high numbers in the families Epistylididae and Vorticellidae and GT4 in Epistylididae/Zoothamniidae and Vaginicolidae.

For Mobilida, we found 442 CAZymes, of which 248 (~56%) were GHs and 109 (~24.5%) were GTs. The most abundant families were GH20, GT2, CE13, GH3 and GT4 acting in structural, microbial cell-wall, unknown and unknown carbohydrates. The representatives of the genus *Trichodina* showed a large number of CAZyme families acting on chitin. *T. acuta* showed the larger number of chitin specialized families among all trichodinids analysed, 7 families (GH18, CE4, GH19, AA11, GT33, GH23 and AA7), *T. hypsilepis* was second in the number of chitin related families, 5 families (AA7, AA11, CE4, GH18 and GH19), and lastly for *Trichodinella* sp*.* with three families found (AA11, GH18 and GH19).

## Discussion

### Genomic features of peritrich ciliates

We found a significant variation in assembly size among the 26 Oligohymenophorea genomes and transcriptomes available in the databases used in this study, ranging from 18.35 to 146.79 Mb. The number of contigs recovered also varied substantially, from 325 to 70,672. Additionally, the genomes sequenced in this study exhibited variation; the size of the seven peritrich genomes from southeastern Brazil ranged from 29.32 to 85.02 Mb, with the recovery of 2,827 to 79,758 contigs per genome and completeness levels between 45.6 and 73.1%. The variation observed in the sequenced genomes is probably related to the biological characteristics of the species analysed. The species selected for this study are particularly challenging to isolate, as most are epibionts closely associated with host organisms. This intimate association often results in unavoidable co-isolation of host material, making the extraction of pure ciliate DNA difficult and potentially introducing contaminant sequences into the datasets. Consequently, the final genome assemblies may present lower completeness scores in standard evaluations such as BUSCO. Nevertheless, the documentation and sequencing of these non-model species are essential steps toward expanding our understanding of ciliate diversity and evolution.

### Orthogroups analyses and phylogenomic reconstruction

The resulting phylogenomic topology is consistent with recent studies based on both phylogenomic and gene-level analyses. The subclass Peritrichia was recovered as monophyletic, presenting a low number of shared orthogroups (128), suggesting genetic specificity among its internal groups. The order Sessilida exhibited 176 shared orthogroups, with 2 exclusives to the clade. In contrast, Mobilida showed 628 shared orthogroups, including 23 specific ones. These findings indicate greater genetic homogeneity in Mobilida; however, this should be interpreted with caution due to limited sampling (only three genomes/transcriptomes). The higher number of specific orthogroups in Mobilida compared to Sessilida may also suggest a higher degree of genetic specialization. This pattern can be attributed to the distinctive characteristics of this order. Mobilids are markedly different from sessilids, particularly due to their unique morphological traits, such as the presence of a permanent swarmer phase and the complex structure supporting the adhesive disc present in all members of the group (28). Thus, the high number of shared orthogroups in these groups is likely linked to these conserved features. Conversely, the lower number of shared orthogroups in Sessilida may reflect the broader morphological diversity within the group.

The internal relationships recovered for the order Sessilida are in line with previous phylogenetic reconstructions. The family Operculariidae was identified as the sister group to all other sessilids, sharing 2,868 orthogroups, 836 of which are family-specific. This high number of shared orthogroups is likely due to the proximity of the two genomes analysed, both belonging to the same genus.

The family Vaginicolidae was identified as the sister group to a clade comprising the families Epistylidae, Vorticellidae and Zoothamniidae. The newly sequenced species, *Platycola decumbens*, emerged as the sister taxon to all other representatives of its family, suggesting substantial divergence consistent with its known distinct lorica morphology [36]. The new genome for the genus *Thuricola* was grouped with its congener. Vaginicolidae exhibited the second highest number of shared orthogroups (507), but only two specific ones, indicating high genetic homogeneity among its members. This pattern is consistent with the conserved production of lorica across species in this family [2,3], which may explain the elevated number of shared orthogroups.

The clade comprising Vorticellidae was recovered as the sister group to the clade containing Epistylidae and Zoothamniidae. This grouping also included *Epistylis anastatica* and *Telotrochidium* sp., a topology supported by previous genetic studies [37,38], and indicative of a closer relationship between these taxa and Vorticellidae. For Vorticellidae, 280 shared orthogroups were identified, none of which were specific to the family – the lowest specificity observed – suggesting greater genetic diversity within this group.

In our analysis, the family Epistylidae was not recovered as monophyletic, as indicated by the placement of *Epistylis anastatica*. Another representative of the family was positioned within the Zoothamniidae clade, which was recovered as the sister group to Epistylidae, corroborating previous findings on the close relationship between these families [1,37]. The clade formed exclusively by Epistylidae species shared 1,531 orthogroups, including 38 specific ones. Regarding Operculariidae, the high number of shared orthogroups likely reflects the close taxonomic relationship of the two analysed genomes.

The clade composed of Zoothamniidae and the Epistylidae representative shared 870 orthogroups, 38 of which were specific. This substantial overlap reinforces the close relationship between the two families and highlights the need for further studies to clarify the evolutionary history of these groups.

### Functional profile of peritrich ciliates

Annotations using the COG database classified over 640,000 genes into 23 categories, mainly related to basic cellular functions. GO database annotations identified over 1 million genes across 113 non-redundant terms, with most falling under ‘Biological Processes’ (25%). KEGG annotations assigned over 400,000 genes to more than 13,000 functional groups, mapping them to 486 pathways, with key pathways involving ‘Metabolism’, ‘Genetic Information Processing’ and ‘Cellular Processes’.

This functional annotation profile reveals significant diversity and complexity in these organisms, demonstrated by the extensive range of annotated COGs, KOs and GOs. The findings also highlight a strong adaptive capacity in the studied ciliates, with a large proportion of genes related to translation, transcription and post-translational protein modification. This suggests an ability to rapidly synthesize and modify proteins in response to environmental changes. Moreover, the abundance of genes linked to metabolic processes points to a high metabolic rate, likely contributing to rapid cell growth and division, further underscoring the ciliates’ adaptive potential.

A total of 15,827 CAZymes were annotated across 476 families. The 33 species analysed had a notably higher number of GHs compared to other CAZyme groups, highlighting the complexity of their energy metabolism, as GHs are essential for breaking down complex carbohydrates for energy. A diverse range of GHs was identified, targeting both structural and non-structural carbohydrates, including microbial cell wall components like peptidoglycan. Many genomes and transcriptomes showed a variety of GHs specific to peptidoglycan degradation, suggesting these organisms are adapted to prey on different bacterial groups. Peritrich ciliates, such as *Epistylis*, *Vorticella* and *Opercularia*, are known bacterivores that help regulate bacterial populations in their environments [14,16, 39]. Although this behaviour is well-documented [2,3, 16, 39], this study provides the first genomic evidence of bacterivory in peritrich ciliates.

In addition to enzymes targeting bacterial cell wall carbohydrates, some organisms also displayed enzymes that act on chitin. The presence of this polysaccharide in peritrich genomes was recently discussed by Jiang *et al*. [10], who reported that chitin is located in different regions of the stalk and cell body of some sessilids. However, information on its occurrence in the order Mobilida is limited. Notably, species from this order – particularly *Trichodina acuta*, analysed in the present study – exhibited genomes with a higher number of CAZyme families specialized in chitin degradation compared to representatives of the order Sessilida. Although Jiang *et al*. [10] demonstrated the importance of chitin in the cellular structure of sessilids, the greater abundance of chitinases in mobilids suggests a possible adaptation to feeding on fragments of their hosts’ integuments, as these ciliates commonly parasitize microcrustaceans whose exoskeletons are primarily composed of chitin [40]. This raises questions about the interaction between *Trichodina* ciliates and their hosts, as *Trichodina –* though typically considered a harmless parasite – can cause damage when present in high concentrations by removing mucus and other protective layers from the host’s tegument through ciliary beating [21,41]. If these ciliates are capable of degrading chitin, the potential harm to the host may be greater than previously thought, as they could cause small abrasions on the tegument [21], potentially opening pathways for secondary infections. While no similar evidence was found in *Trichodina* species parasitizing fish, if confirmed in other groups, this ability could have significant implications, particularly in commercial fish farming [21,42].

Although GHs were the most abundant CAZymes across all organisms analysed, members of the family Vaginicolidae showed a high concentration of GTs, enzymes involved in glycoprotein synthesis (Fig. 1). This suggests these organisms have an enhanced ability to synthesize glycoconjugates, likely related to the presence of the lorica, a key feature in this family [2]. The lorica is composed of glycosaminoglycans and a protein-polysaccharide complex known as pseudochitin or tectin [43], though its synthesis mechanism is poorly understood. Recently, Zhang *et al*. [14] identified GO terms specific to glycosaminoglycan and chitin in Vaginicolidae through GO enrichment analysis, providing further evidence of gene functions linked to lorica formation. A deeper analysis of GTs in these organisms, along with broader sampling within the Vaginicolidae family, could clarify the synthesis of these important structures.

## Conclusions

The analyses revealed significant functional diversity among peritrichs, as indicated by the large number of genes annotated across various categories and functional domains. EggNOG annotations showed a predominance of genes related to post-translational modification, translation and energy metabolism, pointing to a high adaptive and metabolic capacity in the studied species. The diversity of CAZymes, particularly GHs, suggests metabolic complexity and the ability to degrade carbohydrates, crucial for environmental adaptation and predation on various bacterial groups. The presence of chitin-degrading enzymes in species like *Trichodina acuta* suggests complex ecological interactions with hosts, with potential implications for natural ecosystems and commercial hatcheries. The high concentration of GTs in the Vaginicolidae family suggests an enhanced capacity for glycoconjugate synthesis, possibly linked to the formation of the lorica. These findings offer a deeper understanding of the complexity and adaptability of peritrich ciliates, indicating that further research into the specific roles of these enzymes and genes could uncover new insights into their ecology and evolution.

## Supplementary material

10.1099/mgen.0.001472 Uncited Supplementary Material 1.

10.1099/mgen.0.001472 Uncited Supplementary Material 2.

## Abbreviations

AAs: auxiliary activities
CAZymes: carbohydrate-active enzymes
CBMs: carbohydrate-binding modules
CEs: carbohydrate esterases
COGs: Cluster of Orthologous Genes
GHs: glycoside hydrolases
GO: gene ontology
GTs: glycosyl transferases
KEGG: Kyoto Encyclopedia of Genes and Genomes
NCBI: National Center for Biotechnology Information
SRA: Sequence Read Archive
