# Supplementary material for: Comparative genomics reveals details into the metabolism of peritrich ciliates (Ciliophora, Oligohymenophorea and Peritrichia)
Source: Microb Genom. 2025 Sep 4;11(9):001472. doi: 10.1099/mgen.0.001472 (PMC12411067; doi:10.1099/mgen.0.001472)

Supplementary Material 1. Map of the sampling sites for the seven peritrich sequences analyzed in this study. Note that only six points are shown, as two species were collected from the same location.

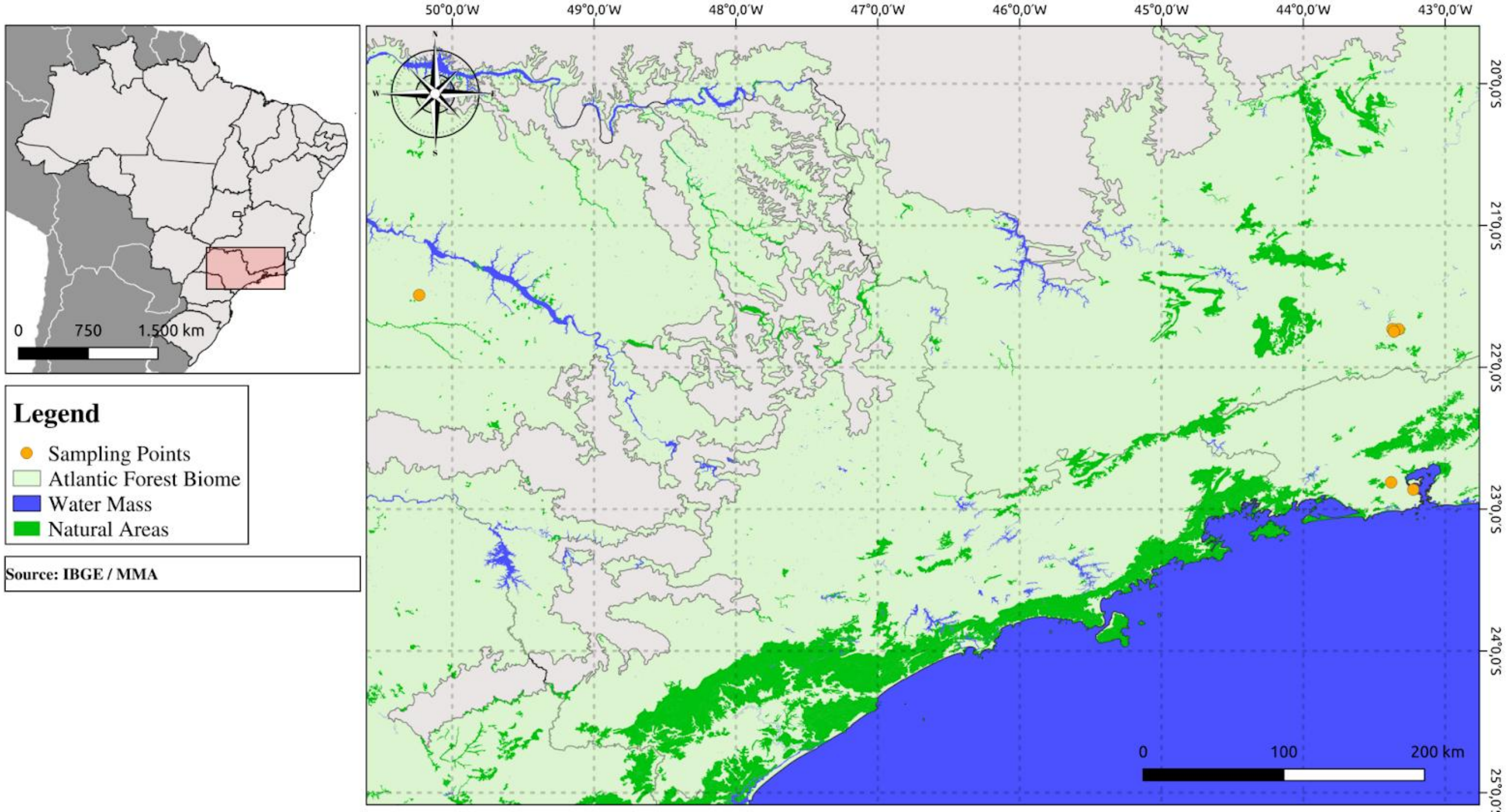

Supplementary Material 2. Table listing the accession numbers for all genomes and transcriptomes used in this study, sourced from the SRA (Sequence Read Archive) database.

**Species**	**SRA Code**
Carchesium polypinum	SRR1768437
Cothurnia ceramicola	SRR12599220
Epistylis anastatica	This work
Epistylis sp. 1	SRR6486645
Epistylis sp. 2	SRR6486646
Epistylis sp. 3	SRR23937120
Myoschiston sp.	SRR6486643
Opercularia sp.	SRR6486644
Opercularia sp. 2	SRR23937118
Paramecium bursaria	GCA_016759055
Paramecium caudatum	GCA_000715435
Paramecium tetraurelia	GCA_000165425
Platycola decumbens	This work
Pseudovorticella sp.	SRR6486649
Telotrochidium sp.	This work
Tetrahymena borealis	GCA_000260095
Tetrahymena malaccensis	GCA_000231845
Tetrahymena thermophila	GCA_016584475
Thuricola similis	This work

Thuricola sp. 1	SRR6486650
Thuricola sp. 2	SRR23937119
Trichodina acuta	This work
Trichodina hypsilepis	SRR6486647
Trichodinella sp.	SRR6486648
Vaginicola sp.	SRR12599219
Vorticella campanula	SRR6486651
Vorticella microstoma	SRR6486652
Vorticella sp. 1	SRR10512982
Vorticella sp. 2	SRR23937121
Vorticella sp. 3	This work
Zoothamnium arbuscula	SRR6486653
Zoothamnium parahiketes	This work
Zoothamnium sp.	SRR6486654

Supplementary Material 3. Graphs displaying the COG (Clusters of Orthologous Groups) functional annotations for each species analyzed, as identified using EggNOG, mapped onto the phylogenomic tree.

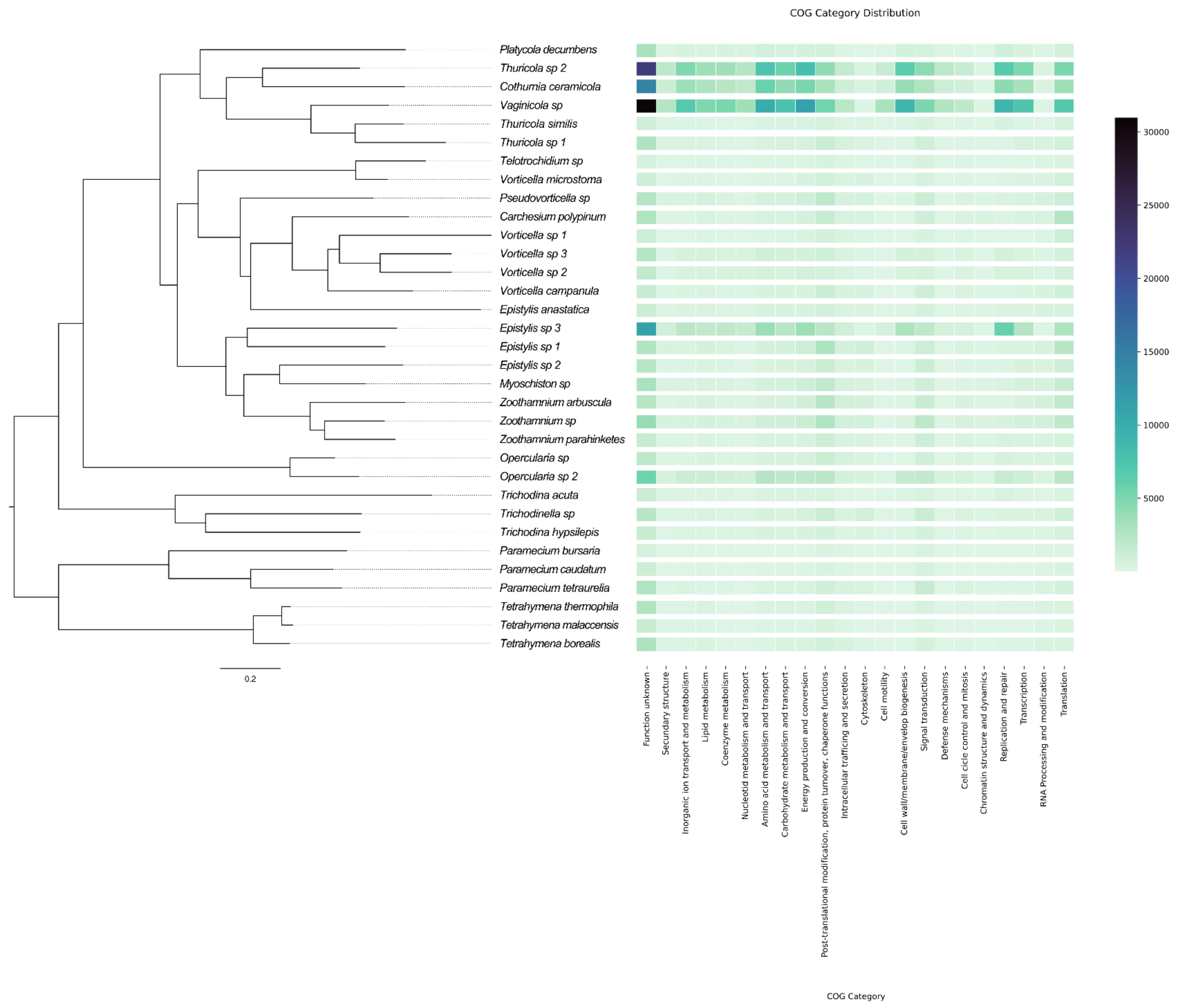

Supplement: Uncited Supplementary Material 1. [file mgen-11-01472-s001.pdf]
